# Successful Surgical Management of Unusual Gallbladder Anatomy Through Laparoscopic Cholecystectomy of Ectopic Gallbladder

**DOI:** 10.7759/cureus.19884

**Published:** 2021-11-25

**Authors:** Mohannad Al-Tarakji, Rashad AlFkey, Hesham Aljohary, Mohammad Sameer, Syed Muhammad Ali

**Affiliations:** 1 Acute Care Surgery, Hamad Medical Corporation, Doha, QAT; 2 Surgery, Weill-Cornell Medical School, Doha, QAT; 3 Acute Care Surgery, Hamad General Hospital, Doha, QAT

**Keywords:** gallstone cholecystitis, surgical management, laparoscopic cholecystectomy, gallbladder, supra-hepatic, ectopic

## Abstract

Abnormalities in the position of the gallbladder are not encountered commonly such as the ectopic location. We present a case of laparoscopic cholecystectomy for gallbladder that was found in an ectopic position. The surgical procedure can be difficult in some cases of acute cholecystitis and ectopic position of the gallbladder may add to complexities of the procedure due to abnormal location or anatomical variants of the biliary tree. Preoperative identification of ectopic gallbladder may aid in planning and performing a safe surgical procedure.

## Introduction

The ectopic gallbladder is one of the rare anatomical anomalies related to the abnormal position of the gallbladder and/or biliary system [1]. Different locations are reported in the literature and those ectopic findings do increase the chances of pathogenicity due to biliary stasis and torsion with the possibility of herniation through the foramen of Winslow [1]. Preoperative diagnosis is crucial in managing and planning treatment to maintain the safety of the patients, arranging for proper instruments, and of great importance to reduce the added risk of possible complications.

## Case presentation

**Figure 1:**
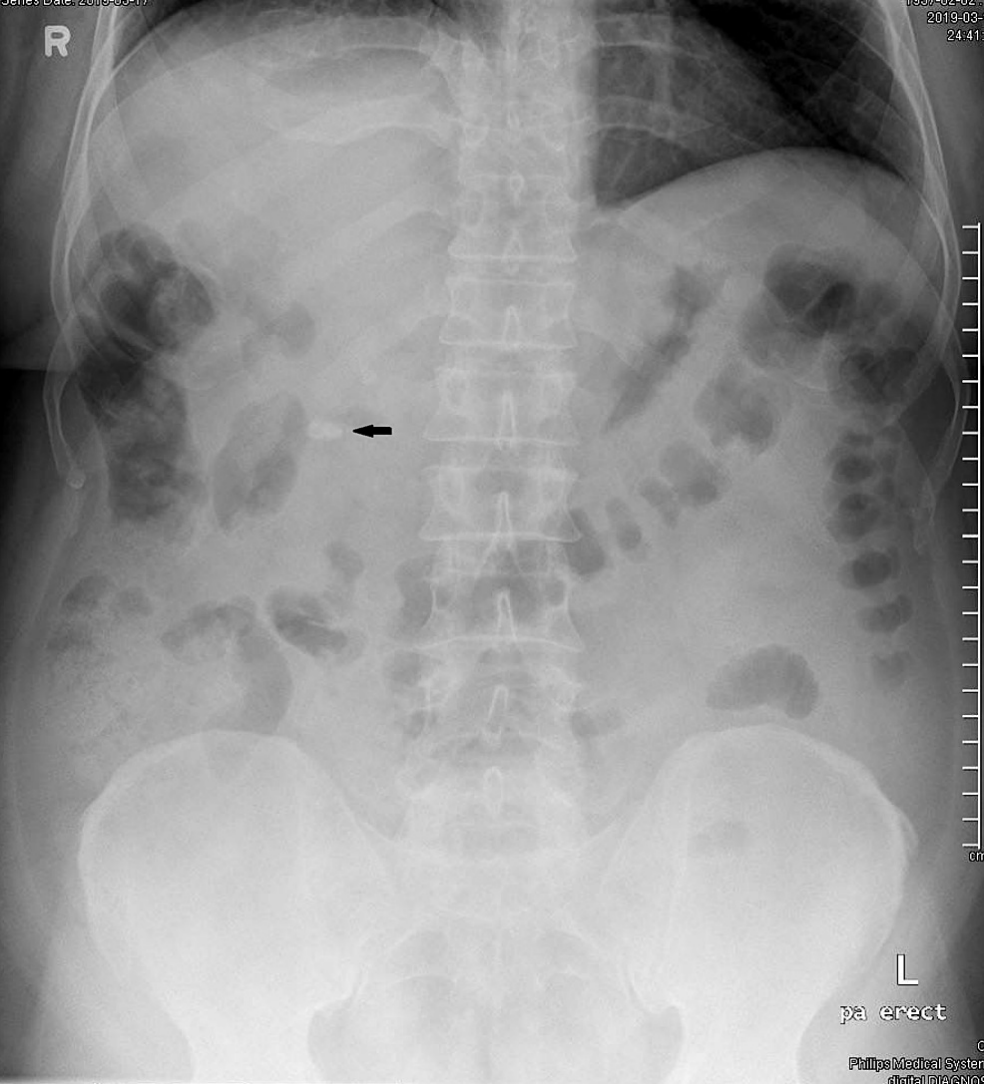
Plain abdominal x-ray showing the radio-dense shadow (black arrow) in the right upper quadrant of the abdomen. Colonic shadow is higher than usual.

A 62-year-old male with a previous history of hypertension, asthma, and obesity with a body mass index (BMI) of 35.6 presented to our Emergency Department with a one-day duration of right upper quadrant abdominal pain associated with nausea and constipation. He had mild pain for the last two days that spontaneously resolved. Vitally he was mildly tachycardic with a heart rate of 96 beats per minute and febrile with a temperature of 38.1⁰C and his laboratory tests were as follows: WBC 15.9/mm^3^, Hemoglobin 13.6g/dL, with all liver functions were within normal limits. X-ray abdomen showed radio dense shadow in the right upper abdomen (Figure 1).

**Figure 2:**
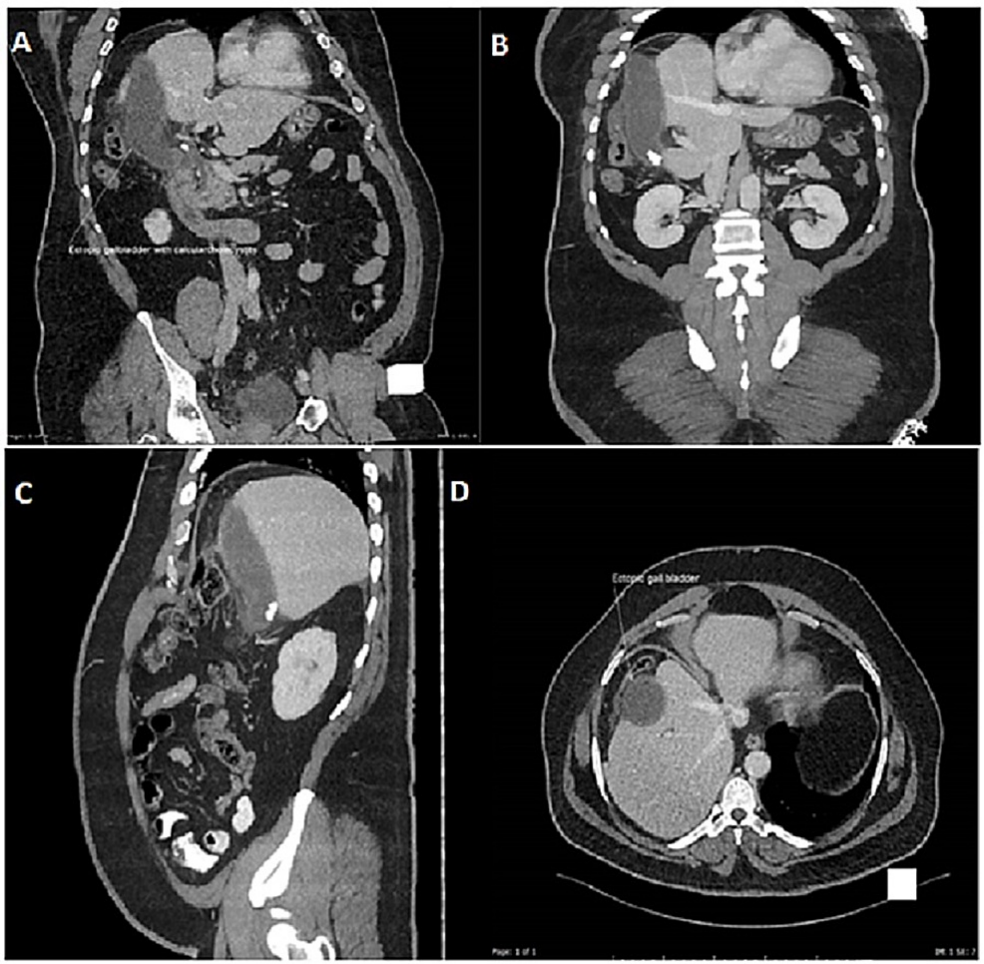
Contrast-enhanced CT scan of the abdomen in axial (A, B), sagittal (C), and coronal (D) views demonstrating the unusual location of gallbladder located lateral to the right lobe of liver with associated features of acute calculous cholecystitis. Partial hypoplasia of the right liver lobe and mild eventration of the right hemidiaphragm is also demonstrated.

Computed tomography (CT) abdomen showed an ectopic gallbladder with multiple gallstones and pericholecystic fluid (Figures 2A-2D).

Ultrasound abdomen showed the liver measuring 16.2cm in length with normal echogenicity. No focal lesion or intrahepatic bile duct dilatation was seen. The body and fundus of the gallbladder demonstrated mild wall thickening with a trace of pericholecystic fluid. The neck of the gallbladder was not visualized due to bowel gas artifact. The common bile duct and portal vein were obscured by bowel gases.

**Figure 3:**
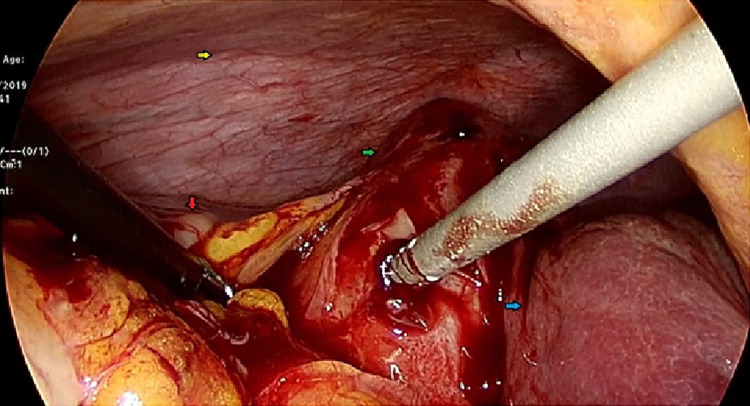
Intraoperative laparoscopic picture showing abnormally located gallbladder (green), liver (blue), eventration of the right diaphragm (yellow), and ascending colon (red).

The patient was admitted and assessed by the anesthesia team as ASA III-E as we planned for laparoscopic cholecystectomy. Cholecystectomy with intraoperative cholangiogram (IOC) was performed with a finding of the right lobe of the liver and gallbladder were located in the chest as the patient has elevated right hemidiaphragm with no diaphragmatic hernia. The gallbladder was located lateral, in ectopic position, to the right lobe of the liver, i.e. from the chest wall lateral to medial were the colon, gallbladder, and then the liver (Figure 3).

**Figure 4:**
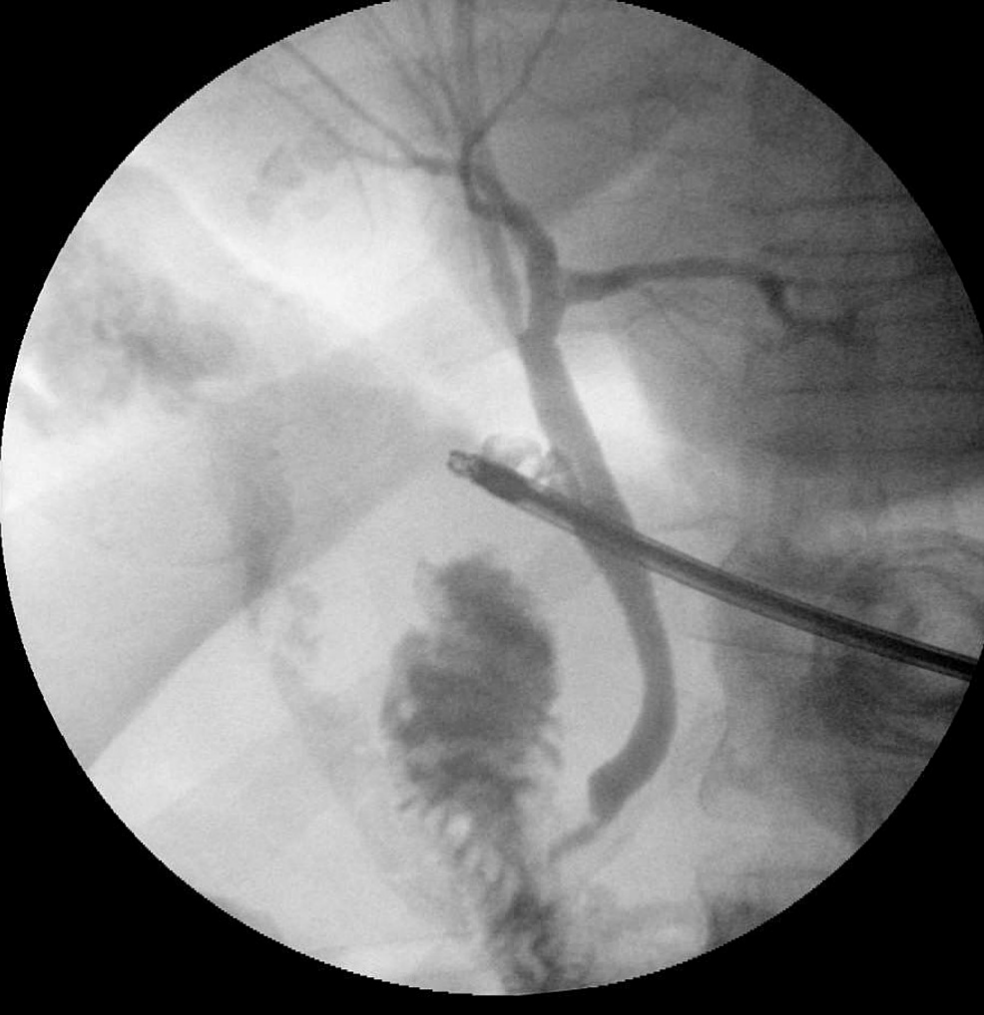
Intraoperative cholangiogram showing normal biliary ductal anatomy.

The gallbladder showed acute chronic inflammation along with extensive adhesion between it, colon, and omentum and dilated cystic duct of around 1 cm. Hemorrhagic fluid was noticed above the liver that was aspirated. The dissection of the gallbladder was commenced on the lateral peritoneum and Calot’s triangle was exposed to isolate the cystic duct and IOC was performed to clarify the anatomy of the biliary tract and showed the normal passage of contrast with no filling defect to the duodenum (Figure 4).

The patient was followed postoperatively for four days where he remained stable, ambulated, tolerated diet and pain, and was discharged home in good condition. Follow-up in surgical outpatient clinic revealed full recovery and histopathology showed acute cholecystitis with cholelithiasis.

## Discussion

Cholecystectomy is one of the most commonly performed abdominal surgical procedures, and in most countries is performed laparoscopically. This procedure is performed for a variety of pathologies like gallstones, polyps and is less likely for gallbladder malignancies.

The ectopic gallbladder as a term is related to the atypical position of the gallbladder with incidence ranging between 0.1% and 0.7% [1]. The gallbladder evolves during in embryo from the foregut through the hepatic diverticulum. Common congenital anomalies of the gallbladder are several and recognized. Infrequent anomalies of the gallbladder are reported also in literature such as agenesis [2], duplication [3,4] septation [5], and Phrygian cap [6]. Adding to the previous anomalies unusual gallbladder position was reported where it might be supra-hepatic in a position [7], left-sided where it is situated on the left side of the ligamentum teres [8], or even floating where it is suspended by mesentery and is free to move [9,10].

Ectopic gallbladders can have significant complications, including a higher incidence of co-existent cholelithiasis [11] due to biliary stasis, risk of torsion if it is suspended on a mesentery, or even herniation through the foramen of Winslow. The possibility of bile duct injury might be higher during cholecystectomy in the ectopic gallbladder [12,13]. Thus, accurate preoperative localization of the gallbladder is of great significance to plan a safe surgical approach and to avoid complications in these rare circumstances.

In a pre-operative era, CT scan and MRCP are the best diagnostics modalities to clarify biliary tree anatomy and abnormalities while sonographic diagnosis might be tricky [14]. The most common cause of operative biliary tree injuries is improper recognizing of the anatomy of the biliary tree. IOC had been used in the past for the detection of biliary injuries; however, recently for real-time identification of biliary anatomy and to avoid injuries, the fluorescent cholangiography by fluorescent agents excreted in the biliary system is performed [15].

Laparoscopic surgery is a safe procedure to be performed in ectopic gallbladder cases. While more complications might be expected with associated extra anomalies of the biliary tree, no data reported the rate of complication due to the rarity of these abnormalities [16].

## Conclusions

Ectopic gallbladder disease is rare pathology but important to be diagnosed pre-operatively if surgery is indicated. Surgical planning by advanced imaging like CT or MRCP is needed if there is suspicion on routine ultrasound examination. An operative procedure should be performed by an experienced surgeon to decrease the chance of biliary injury with aid of IOC or fluorescent cholangiography, if available.
